# Vascular mild cognitive impairment and its relationship to hemoglobin A1c levels and apolipoprotein E genotypes in the Dominican Republic

**DOI:** 10.1590/1980-57642021dn15-010007

**Published:** 2021

**Authors:** Martin Medrano, Gelanys Castro-Tejada, Rafael Lantigua, Gretel Silvestre, Sergio Diaz, Patricia Mota, Franck Diaz-Garelli

**Affiliations:** 1 School of Medicine, Pontificia Universidad Católica Madre y Maestra – Santiago, Dominican Republic.; 2 Biomedical and Clinical Research Center, Hospital Universitario José Maria Cabral y Baez – Santiago, Dominican Republic.; 3 The Taub Institute for Research on Alzheimer’s Disease and the Aging Brain – New York, NY, USA.; 4 Department of Medicine, Columbia University, The New York Presbyterian Hospital – New York, NY, USA.; 5 Neuroscience and Behavior Research Program, Pontificia Universidad Católica Madre y Maestra – Santiago, Dominican Republic.; 6 School of Psychology, Pontificia Universidad Católica Madre y Maestra – Santiago, Dominican Republic; 7 Internal Medicine Service at Hospital Universitario Jose Maria Cabral y Baez – Santiago, Dominican Republic.; 8 Department of Preventive Medicine, Hospital Metropolitano de Santiago – Santiago, Dominican Republic.; 9 University of North Carolina at Charlotte – Charlotte, USA.

**Keywords:** vascular dementia, risk factors, apolipoproteins E, metabolic syndrome, diabetes mellitus, demência vascular, fatores de risco, alipoproteínas E, síndrome metabólica, diabetes mellitus

## Abstract

Dementia and vascular mild cognitive impairment (VaMCI) currently impose a tremendous human and economic burden on patients from aging populations and their families worldwide. Understanding the interplay of cardiometabolic risk factors and apolipoprotein E (APOE) may direct us to a more personalized medicine and preventative care in MCI and dementia.

**Objective:**

To evaluate the relationship of cardiometabolic risk factors with MCI and assess the APOE genotype’s role in an elderly cohort in the Dominican Republic.

**Methods:**

We studied a cohort of 180 participants 65 years of age and older using a combined assessment of cardiometabolic risk factors, neuropsychological battery tests, and APOE genotyping. We used the number of failed tests as a proxy to predict MCI.

**Results:**

We found that patients with the ε3-ε4 APOE genotype had 2.91 higher number of failed cognitive tests (p=0.027) compared to patients with the ε3-ε3 genotyped. The rate of test failures increased 10% (p=0.025) per unit increase in HbA1c percentage.

**Conclusions:**

Increased Hemoglobin A1c levels and ε3-ε4 APOE genotypes seem to have an association with the development of VaMCI.

## RESUMO.

A demência e o comprometimento cognitivo leve vascular (VaMCI) atualmente impõem uma enorme carga humana e econômica aos pacientes de populações envelhecidas e suas famílias em todo o mundo. Compreender a interação dos fatores de risco cardiometabólicos e apolipoproteína E (APOE) pode nos direcionar para uma medicina mais personalizada e de cuidados preventivos em MCI e demência.

**Objetivo:**

Avaliar a relação dos fatores de risco cardiometabólicos com o MCI e o papel do genótipo APOE em uma coorte de idosos na República Dominicana.

**Métodos:**

Estudamos uma coorte de 180 participantes com 65 anos de idade ou mais, utilizando uma avaliação combinada de fatores de risco cardiometabólicos, uma bateria de testes neuropsicológicos e genotipagem APOE. Adotou-se o número de testes com mau desempenho para o diagnóstico de MCI.

**Resultados:**

Verificou-se que os pacientes com o genótipo ε3-ε4 do APOE apresentaram 2,91 vezes mais testes cognitivos com mau desempenho (p=0,027) em comparação com os pacientes com o genótipo ε3-ε3. A taxa de falhas de teste aumentou 10% (p=0,025) por aumento de unidade na porcentagem de HbA1c.

**Conclusões:**

Níveis mais altos de HbA1c e os genótipos ε3-ε4 do APOE parecem estar associados ao desenvolvimento de VaMCI.

## INTRODUCTION

Dementia and vascular mild cognitive impairment (VaMCI) currently impose a tremendous human and economic burden on patients from aging populations and their families worldwide.^1^ VaMCI encompasses the cerebrovascular continuum from MCI to dementia,^2^ beginning with cardiometabolic risk factors leading to cerebral vascular disease (CVD) of the large and small arteries. This disease will lead to different types of cognitive impairments depending on the location of the damage produced.^3^ ^–^ ^5^ Associated risk factors comprise the entire cluster of classical vascular risks: Hypertension (HTN), hyperlipidemia, Diabetes Mellitus Type 2 (DM2), metabolic syndrome (MetS), smoking, and age but also the emerging risk factors:^6^ high sensitivity C-reactive protein and homocysteine, behaving as pro-inflammatory markers and prothrombotic status, respectively. When vascular diseases have been associated with MCI, the incidence in patients with at least one vascular disease doubles the frequency of MCI in the healthy population.^7^ HTN also seems to have a strong influence when developed during mid-adulthood as compared to its appearance in an older age.^8^

DM2 has also been associated with executive function impairment, including reasoning, mental flexibility, problem solving, and decision making; especially with high glucose levels, worst performance on executive cognitive tasks was seen when DM2 would onset in midlife.^9^ Evidence for the relationship between diabetes and vascular dementia has been consistent,^10^ both the American Diabetes Association (ADA) and the American Heart Association (AHA) have stated the need to develop reliable estimates of the combined cardiometabolic risks for patient care.^11^ Metabolic syndrome, as well, encompasses multiple coexisting factors associated with cognitive function decline in elderly populations.^12^ ^,^ ^13^ However, most cardiovascular risk factors are modifiable; genetics are also likely to play a role.^14^ Understanding this interplay may unlock the promise of personalized medicine and preventive care in MCI and dementia.^15^ The presence of at least one allele of apolipoprotein E (APOE) ε4 is a risk factor for cardiovascular diseases and increases the risk of dementia in subjects who have suffered a stroke,^16^ ^,^ ^17^ being particularly pronounced factors in the Latino population.^18^ APOE is involved in lipid metabolism and can modulate the relationship between vascular factors, diabetes, and cognitive functions.^19^ ^,^ ^20^

The geriatric neuropsychological battery (NPB) is a great tool to identify patients with MCI at risk of progressing to dementia.^21^ In conjunction with NPB, early identification of vascular risk factors and treatment of associated diseases may decrease vascular cognitive impairment incidence.^17^ However, the literature is still unclear about the interplay between APOE genotypes, cardiometabolic risk factors, and cognitive impairment. This research group investigated cardiometabolic risk factors to address this gap, aiming to convey findings that may lead to the development of clinical protocols for early prevention, diagnosis, and treatment of cognitive changes in aging patients. The objective of this study was to evaluate the relationship of cardiometabolic risk factors with MCI and to assess the role of the APOE genotype in an elderly cohort in the ethnically heterogeneous Dominican Republic.

## METHODS

### Study description

The Cardiometabolic, Cerebral and Genetic Factors and Their Influence on Neurocognitive Functions and Depression in the Elderly (CEGENED) study, was conducted in Santiago, Dominican Republic. The cohort comprised 180 subjects aged 65 years old or older, both male and female. The study period lasted four years. For this paper, the first assessment, performed between July 2014 and May 2015, was analyzed. The purpose of the study and the participants’ role were written, and verbally explained, following the evaluation of the participants’ comprehension of its benefits and consequences. They proceeded to sign the consent form, per protection of human subject standards. Participants were invited from primary care consultations to community gatherings, where objectives of the study were explained. An initial interview assessed demographic variables, including age and gender, and self-reported level of education. During the visit, an in-depth medical history was also conducted, in addition to a biophysical medical evaluation, Columbia University validated neuropsychological battery tests,^22^ and a Depressive Symptoms (Center for Epidemiologic Studies Depression Scale — CES-D) test.^23^ Smoking for more than a month and any history of alcohol intake were self-reported.

Inclusion criteria were:

- Dominican citizens aged 65 years old or older;
- No history of CVD or depression;
- Being able to perform basic and instrumental activities of daily living autonomously (Lawton and Brody Scale,^24^ and Barthel Index).^25^
- Being able to use public transportation independently and apparent normal cognition (short Blessed test^26^ score).

Exclusion criteria were:

- History or evidence of stroke.
- Myocardial infarction.
- Neurological or psychiatric conditions such as psychotic disorders or bipolarity.
- Alcoholism (intake greater than 30 gr a day for men and 20 gr a day for women).
- History of brain surgery.

This study was approved by the Committee of Bioethics of the Pontificia Universidad Católica Madre y Maestra (COBE-FACS) and the National Committee on Bioethics (CONABIOS): 002-2-2013-2014.

### Cardiometabolic evaluation

Cardiometabolic factors were assessed using the diagnostic criteria described in the AHA guidelines.^27^ DM was considered in anyone with prior diagnosis or who is currently being treated with glucose-lowering medication. Diagnosis would be made if there was no history of diabetes, but a fasting glucose level greater than 126 mg/dL or HbA1c level greater than 6.5%. Hypertension is defined as systolic blood pressure ?130 mmHg and diastolic blood pressure ?80 mmHg. Diagnosis was made according to the 24-hour Ambulatory Blood Pressure Monitoring report for anyone with prior diagnosis or currently treated with antihypertensive medications, or HTN diagnosis status unknown. The lipid profile was determined using standard enzymatic techniques. High-density lipoprotein cholesterol (HDL-C) was determined using a cholesterol laboratory standard method for CVD biomarkers. High sensitivity C-reactive protein was measured using an ultrasensitive enzyme-linked immunosorbent assay. Criteria of the Third Report of the National Cholesterol Education Program Expert Panel of Detection, Evaluation and Treatment of High Blood Cholesterol in Adults (Adult Treatment Panel III), were used to determine the metabolic syndrome if three or more of the following criteria coexisted: Abdominal obesity determined by a waist circumference greater than 102 cm in men and 88 cm in woman, triglycerides greater than or equal to 150 mg/dL, HDL-C lower than 40 mg/dL in men and 50 mg/dL in women, HTN diagnosed previously or by the Holter during the study, and diabetes previously diagnosed or an HbA1c greater than 6.5%. Homocysteine levels were determined by the Abbott homocysteine (HCY) assay considering normal values of up to 15 μmol/L. APOE genotyping was performed at Columbia University in the Taub Institute for Research on Alzheimer’s Disease and the Aging Brain using a polymerase chain reaction-based method. APOE genotype was determined by Polymerase Chain Reaction-Restriction Fragment Length Polymorphism (PCR-RFLP) using the enzyme HhaI.^28^ ^–^ ^30^

### Neuropsychological assessment

All participants were cognitively normal according to the initial Short Blessed Test assessment. The neuropsychological battery^22^ was then applied, including the following tests and their cut-off values:

- Memory (Selective Reminding Test total recall <25, Selective Reminding Test long-term recall <15, Selective Reminding Test delayed recall <4, Delayed recognition <8, Benton Visual retention Test multiple choice recognition <7).
- Orientation <8.
- Construction (Benton Visual retention test multiple-choice matching <7).
- Abstract reasoning (Mattis identities and oddities <12).
- Language (Boston Diagnostic Aphasia Evaluation repetition of high probability phrases <7, Boston Diagnostic Aphasia Evaluation complex ideation material <5, Boston Naming Test <11).
- Controlled Oral word association <16 percentile.

Subject differences were explored along two dimensions: patients who scored below the tests’ cutoff values classified as MCI patients *versus* not along with the raw number of failed cognitive tests per patient. All participants underwent brain magnetic resonance imaging (MRI) to determine vascular brain damage status.

### Imaging techniques

Noninvasive cardiologic and brain imaging studies included: Electrocardiogram and echocardiogram, brain magnetic resonance with Brain MRI (Toshiba 1.5 tesla): Coronal section cuts of 0.3 mm. Axial and sagittal section cuts of 4 mm in T1, T2, and FLAIR. Also, white matter hyperintensities and brain atrophy were assessed by the FAZEKAS visual scales. Anterior and posterior extracranial circulation was evaluated by carotid Doppler using Mindray M7 Duplex Scan equipment of high-resolution 3D/4D color images, B-mode, with 7L4sMHZ transducer. Thickening of the medial intima greater than or equal to 1.0 mm was considered stenotic and associated with atherosclerosis.

### Diagnosis of vascular mild cognitive impairment

The diagnosis of VaMCI was based on the new criteria proposed by the International Society for Vascular Behavioral and Cognitive Disorders (VASCOG)^31^ comprising two main categories:

- The cognitive deficits are not sufficient to interfere with independence (*i.e*., instrumental activities of daily living are preserved), but greater efforts, compensatory strategies, or accommodation may be required to maintain independence.
- Determining that vascular disease is the dominant or exclusive cause of cognitive deficits.

Cognitive performance impairment in neuropsychological assessment was considered when between 1 and 2 standard deviations below the norm, or between the 3rd and 16th percentile in individuals of similar age, gender, education, and sociocultural backgrounds. One of the exclusion criteria was the history of known stroke. Subjects with large vessel disease or atherothrombotic diseased patients were also excluded. For the deemed VaMCI, we have considered silent infarcts, small vessel disease: multiple lacunar infarcts in the white substance or the gray matter of the basal nuclei, extensive and confluent vascular leukopathy (Fazekas II-III scale), microinfarcts, and cortical microhemorrhages.

### Statistical analysis

For this analysis, the number of test failures were assessed as a proxy to predict the intensity of VaMCI in each patient, as done in previous studies.^32^ ^,^ ^33^ A test was considered failed when its cognitive test score was below the cut-off value. We explored initial associations with univariate models predicting VaMCI in patients with a binomial regression and the number of failed tests with count regressions. Multivariate count regressions were built to predict the number of neuropsychological tests failed using R’s generalized linear model (GLM).^34^ We tested our datasets for zero inflation using the Van den Broek’s zero-inflation test^35^ and for overdispersion by comparing means and standard deviations in our primary outcome variable (*i.e*., number of failed tests). We selected classic Poisson regressions to fit our non-zero-inflated, non-over-dispersed data. For all the models, the number of failed tests was this study’s primary outcome variable. Models were built using a forward selection process. Specifically, there was a pre selection of variables based on predictive power according to Harell’s variable selection approach,^36^ setting the p-value variable inclusion threshold at 0.1. Then, each pre-selected variable’s relationship to VaMCI and APOE genotypes were explored. The Akaike’s information criterion was used to select the final model by maximizing each model’s goodness of fit.^37^ All models were tested for variable interactions and collinearity effects with more than one variable. Adjustments for multiple comparisons were made using R’s p. adjust function, selecting Holm’s correction method.^38^ Statistical significance was set at p=0.05 for all models.

## RESULTS

180 participants were recruited, two died before completing data collection, and two were lost to follow up. This study’s final cohort contained 176 participants, with 70.5% of the total sample affected by Non-VaMCI (Table 1). Age ranges from 65 to 77 years, with 30.11% males and 69.3% females. 58.86% of patients had a history of HTN, 25.5% had a history of diabetes, and 68.18% completed primary education. Mean systolic blood pressure was at stage I hypertension (153.4 mmHg), but the mean diastolic blood pressure was normal (78.7 mmHg). Mean cholesterol was (198.42 mg/dL), mean triglycerides was (138 mg/dL), and mean low-density lipoprotein cholesterol (LDL-C) was (122.6 mg/dL), while the mean glycated hemoglobin was 6.6%, characterizing diabetes. Exploring group differences and statistically significant relationships between our variables and VaMCI groups, no statistically significant relationships were observed, likely due to the limited cohort size.

**Table 1:** Demographics, cardiometabolic risk factors, genetic, and neuropsychological assessments.

Characteristics			Non-VaMCI (n=127)	VaMCI (n=49)	p-value
Age (years)			71±6.03	73±6.28	0.863
Female (%)			88 (69.3)	35 (71.4)	0.0649
Education (years)			5.49±3.31	4.95±2.67	0.992
Diabetes mellitus			31 (24.4%)	12 (24.5%)	0.901
Hypertension			70 (55.1%)	33 (67.3%)	0.0996
Systolic blood pressure (mmHg)			153.39±24.88	154.47±26.62	0.189
Diastolic blood pressure (mmHg)			78.68±10.67	79.80±11.81	0.568
MAP (mmHg)			100.53±16.60	99.06±22.29	0.480
BMI (kg/m^2^)			27.6±6.99	26.29±3.96	0.413
Waist circumference (cm)			95.1±12.3	97.06±9.96	0.457
Total cholesterol (mg/dL)			198.42±41.71	196.28 ±40.96	0.968
Triglyceride (mg/dL)			138±80.84	138.69±63.22	0.980
LDL-C (mg/dL)			122.6±36.7	122.5±36.4	0.715
HDL-C (mg/dL)			46.66 ±10.85	45.63±10.75	0. 646
HbA1c (%)			6.56±1.18	6.69±1.18	0.619
C-reactive protein			5.50±8.06	4.39 ±6.12	0.217
Homocysteine			17.9±6.77	19.7±9.46	0.856
Smoking			55 (47.02%)	20 (45.26%)	0.562
MetS			54 (49.09%)	21 (48.84%)	0.348
CES-D			92 (83.64%)	14 (12.73%)	0.490
Neuropsychological Battery					
	Memory				
		SRT total recall	37.69±9.69	36.24±9.15	0.373
		SRT long-term recall	24.96±12.55	23.26±10.93	0.451
		SRT delayed recall	5.48±2.35	5.59±2.28	0.709
		Delayed recognition	11.19±1.30	11.16±1.32	0.991
		BVRT multiple-choice recognition	5.60±2.30	5.18±2.26	0.338
Orientation			8.16±1.40	8.02±1.24	0.571
Construction					
	BVRT multiple-choice matching		7.31±2.34	7.26±2.06	0.950
Abstract reasoning					
	Mattis identities and oddities		14.90±1.26	14.26±1.61	1
Language					
	BDAE repetition of high probability phrases		7.80±0.50	7.79±0.45	0.756
	BDAE complex ideation material		4.44±1.18	4±1.59	0.071
	Boston Naming Test		13.32±1.70	12.65±2.86	0.105
CFL			24.65±20.65	20.44±18.18	0.202
APOE genotype					
	ε2-ε2		1 (0.79%)		0.996
	ε2-ε3		10 (7.97%)	7 (14.29%)	0.991
	ε2-ε4			1 (2.04%)	0.988
	ε3-ε3		82 (65.08%)	35 (71.43%)	0.992
	ε3-ε4		30 (23.81%)	6 (12.24%)	0.992
	ε4-ε4		3 (2.38%)		0.992
	Indeterminate		1 (0.85%)		1

APOE: apolipoprotein E; VaMCI: vascular mild cognitive impairment; MAP: mean arterial pressure; BMI: body mass index; HDL-C: high-density lipoprotein cholesterol; LDL-C: low-density lipoprotein cholesterol; HbA1c: glycated hemoglobin; MetS: metabolic syndrome; CES-D: Center for Epidemiologic Studies Depression Scale; SRT: Selective Reminding Test; BVRT: Benton Visual Retention Test; BDAE: Boston Diagnostic Aphasia Evaluation; CFL: Controlled Oral Word Association.

Demographic data of all aged patients comparing VaMCI and non-vascular cognitive impairment. MAP: mean arterial pressure; BMI: body mass index; LDL-C: low-density lipoprotein cholesterol; HDL-C: high-density lipoprotein cholesterol; HbA1c: glycated hemoglobin; MetS: metabolic syndrome; CES-D: Center for Epidemiologic Studies Depression Scale; SRT: Selective Reminding Test; BVRT: Benton Visual Retention Test; WAIS-R: Wechsler Adult Intelligence Scale-Revised; BDAE: Boston Diagnostic Aphasia Evaluation; CFL: Controlled Oral Word Association; APOE: apolipoprotein E genotypes. Even though there was genotype variability among participants, we chose to focus on APOE ε3-ε3 and ε3-ε4 due to data availability. Statistical differences were evaluated with univariate logistic regression models predicting patient’s belonging to the VaMCI group or not. Values are mean ± standard deviations or n (%).

Our population presented a high rate of cognitive test failures (*i.e*., a cognitive test score below its cut-off value) for patients with both APOE genotypes of interest (Table 2). We report the overall number of failed tests for each cognitive category tested; therefore, the number of failed tests is higher than the number of patients in the cohort. Construction and Abstract reasoning tests had close to 100% failure rates for both genotypes, whereas language tests showed no differences (67.80 *vs*. 77.78% for ε3-ε3 and ε3-ε4 genotypes, respectively). Nevertheless, we found significant differences for orientation tests (24.58 *vs*. 44.44% for ε3-ε3 and ε3-ε4, respectively). Memory tests, however, presented low failure rates: 6 *vs.* 4 test failures overall, corresponding to 5.08 *vs.* 11.11% failure rates of ε3-ε3 and ε3-ε4, respectively. The mean failure rates were relatively similar, with 4.92+-2.25 and 5.75+-2.26 for APOE genotypes ε3-ε3 and ε3-ε4, respectively. Near-significant group differences were found between ε3-ε3 and ε3-ε4 APOE groups for orientation tests and the overall number of test failures.

**Table 2:** Number of patients with failed cognitive tests and number of test failure. Statistical differences were evaluated with univariate logistic regression models predicting patient’s belonging to each apolipoprotein E group.

Area impaired	APOE ε3-ε3 n=117	APOE ε3-ε4 n=36	p-value
Memory	6 (5.08%)	4 (11.11%)	0.231
Orientation	29 (24.58%)	16 (44.44%)	0.0608
Construction	117 (100%)	36 (100%)	1
Abstract reasoning	117 (100%)	36 (100%)	1
Language	80 (67.80%)	28 (77.78%)	0.280
Overall number of failed tests (% of total tests)	578(32.9%)	207(38.7%)	0.0609

APOE: apolipoprotein E.

Our preliminary cardiometabolic risk factor variable screening based on their relationships with the number of failed cognitive tests revealed statistically significant relationships for mean arterial pressure, waist circumference, weight, and HbA1c (Table 3).

**Table 3:** Statistical association between individual cardiometabolic risk factors and the number of failed cognitive tests using a count model using the full cohort sample.

Term	Rates ratio (exp(ß))	Estimate (ß)	95% confidence interval		Standard error	p-value
Gender	1.053	0.0524	(-0.099,	0.201)	0.076	0.493
Age	1.005	0.0055	(-0.005,	0.016)	0.005	0.335
Hypertension	1.114	0.1080	(-0.033,	0.251)	0.072	0.137
Diabetes	1.089	0.0859	(-0.0827,	0.249)	0.084	0.310
HbA1c*	1.054	0.0533	(-0.0054,	0.109)	0.029	0.069
Waist circumference*	0.747	-0.2905	(-0.545,	-0.035)	0.130	0.025
BMI	0.994	-0.0055	(-0.020,	0.009)	0.007	0.474
Weight*	0.993	-0.0060	(-0.011,	-0.0005)	0.002	0.031
Triglyceride	0.999	-7.90E-05	(-0.001,	0.0008)	0.000	0.870
HDL-C	0.995	-0.0048	(-0.011,	0.001)	0.003	0.147
LDL-C	1.000	0.0007	(-0.001,	0.0026)	0.000	0.428
MAP*	1.132	0.1242	(-0.017,	0.266)	0.072	0.086

HbA1c: glycated hemoglobin; BMI: body mass index; HDL-C: high-density lipoprotein cholesterol; LDL-C: low-density lipoprotein cholesterol; MAP: mean arterial pressure. Variables presenting a relationship with the number of failed cognitive tests are marked with an asterisk. Statistical differences were evaluated with univariate count regression models predicting the number of failed tests for each patient.

This study’s final model predicting overall test failures disclosed statistically significant relationships between the number of failed tests, the APOE genotype, and the patients’ HbA1c levels at the day the tests were administered (Table 4). HbA1c, waist circumference, weight, and MAP were pre-selected for modeling in accordance with the previously defined variables selection strategy^36^ that sets our p-value variable inclusion threshold at 0.1. This model was selected based on our candidate variables (*i.e*., MAP, waist circumference, and HbA1c). Though MAP and waist circumference showed a relationship with the number of failed tests, they failed to show a joint relationship mediated by APOE genotypes (p=0.14 and 0.24, respectively). In contrast, HbA1c presented a relationship with the APOE genotype and evidence of statistically significant interaction, revealing a strong interrelation. Specifically, we found that patients with the ε3-ε4 genotype have a 2.91 rate ratio for failed cognitive tests (Adj-p=0.027) compared to patients with the ε3-ε3 genotype. The number of failed tests would increase by a rate of 10% (Adj-p=0.025) for each one-unit increase in a patient’s HbA1c values. Finally, we found a statistically significant interaction between the APOE genotype and the HbA1c, revealing that the effect of the HbA1c was lower by 13% (Adj-p=0.028) in patients with the ε3-ε4 APOE genotype compare those with the ε3-ε3 genotype. The number of failed tests for patients in the APOE ε3-ε3 genotype group was more susceptible to being affected by HbA1c values than those in the APOE ε3-ε4 group, which already had much higher rates of failed test. We explored the effect of cardiometabolic factors such as prior diagnoses of hypertension and diabetes, weight, BMI, waist circumference and lipid values, and relevant variable interactions during the model building phase. However, it was not possible to find any other statistically significant relationships in multivariate models that included the APOE groups of interest. We would, therefore, provide the appropriate evidence to test our hypothesis.

**Table 4:** Final count regression model predicting the number of overall failed tests for the full patient cohort.

Regression term	Rates ratio (exp(ß))	Estimate (ß)	Confidence interval (95%)		Standard error	p-value	Adjusted p-value
Intercept	2.59	0.95	(0.470,	1.45)	0.249	0.0001	0.0005
APOE ε3-ε3 (Reference)	1	1	-		-	-	-
APOE ε3-ε4	2.91	1.07	(0.228,	1.93)	0.434	0.013	0.027
HbA1c	1.10	0.098	(0.0238,	0.170)	0.037	0.008	0.025
APOE ε3-ε4: HbA1c	0.87	-0.138	(-0.264,	-0.016)	0.063	0.028	0.028

APOE: apolipoprotein E; HbA1c: glycated hemoglobin.

This finding was explored further by predicting the counts of failed cognitive and memory tests rather than overall failures in our patient population (Table 5). Our final model predicting cognitive test failures only included the APOE variables, revealing a 19.4% higher number of failed cognitive tests for patients with the ε3-ε4 genotype compared to patients with the ε3-ε3 genotype (Adj-p<.0001). The univariate model predicting the number of failed cognitive tests based on the HbA1c did not reveal a statistically significant relationship (Adj-p=0.172). Still, the same model developed for the overall number of failed tests returned similar estimate values with near-significance before p-adjustment. For example, the APOE group differences returned a rates ratio of 2.55 (p=0.040) compared to 2.91 (Adj-p=0.027) and the HbA1c returned a rate ratio of 1.08 (p=0.052) compared to 1.1 (Adj-p=0.025). This may be due to the reduced number of tests (*i.e*., cognitive tests only) and may be overcome with additional data. Other cardiometabolic factors were explored to predict the number of failed cognitive tests and relevant variable interactions during model building and development. For this model, we also explored the effect of the cardiometabolic factors stated above and relevant variable interactions. Still, it was not possible to find other statistically significant relationships for the models, including the APOE group variable. The possibility of modeling the number of failed memory tests only was also explored. However, no statistically significant relationships were found for the variables of interest. Our dataset presented zero-inflation (69.3% of patients failed no memory tests), reducing our dataset variability and the potential for reliable statistical modeling.

**Table 5:** Final count regression results predicting the number of failed *cognitive and memory tests* for the full patient cohort.

Model	Regression term	Rates ratio (exp(ß))	Estimate (ß)	Confidence interval (95%)		Standard error	p-value	Adjusted p-value
Full model	Intercept	2.676	0.984	(0.469,	1.515)	0.267	0.0002	0.001
	APOE ε3-ε4 (Ref. 3-3)	2.55	0.936	(0.052,	1.838)	0.455	0.040	0.119
	HbA1c	1.08	0.078	(-0.003,	0.155)	0.040	0.052	0.119
	APOE ε3-ε4: HbA1c	0.892	-0.114	(-0.247,	0.014)	0.066	0.085	0.119
APOE only	Intercept	4.444	1.492	(1.404,	1.576)	0.044	<.0001	<.0001
	APOE ε3-ε4 (Ref. 3-3)	1.194	0.177	(0.009,	0.341)	0.085	0.036	0.036

APOE: apolipoprotein E; HbA1c: glycated hemoglobin.

## DISCUSSION

We found that the number of failed cognitive tests in geriatric patients with cardiovascular risk factors was related to their APOE genotype and their HbA1c levels. Statistically significant differences were observed in the number of failed neuropsychological tests and APOE genotypes ε3-ε3 and ε3-ε4. ε3-ε4 genotype patients seemed to fail more cognitive tests. The HbA1c values at the time of testing appeared to affect the overall number of failed tests, but mostly in ε3-ε3 genotype patients. No HbA1c effect was found for cognitive tests alone.

Previous research established that the APOE gene is associated with the development of Alzheimer disease (AD).^39^ The E allele is also associated to code for the regulation of sugar and lipid metabolism. AD and MCI have a common pathway with diabetes and other metabolic disorders that could influence mechanisms for deterioration of cognitive functions.^40^ Significant positive association has been found in APOE ε4 carriers, increasing the risk of developing vascular dementia as compared to ε2 and ε3 carriers.^41^ Our findings showed further differences in APOE alleles, underscoring an increased number of test failures in the ε3-ε4 as compared to the ε3-ε3 group. Furthermore, the length of time patients have diabetes is associated with greater cognitive decline after 20 years; this association is dependent on HbA1c levels, and greater cognitive decline observed with HbA1c >7%.^9^ Non-adherent diabetics also had worse performance in executive functions, memory tasks, and mental planning.^42^ The impairments were accentuated in cognitive domains such as information processing speed, executive function,^9^ memory loss,^43^ and visual construction skills, suggesting diabetes damages microvasculature of the subcortical gray matter or other pathways. Similarly, we found a strong association between HbA1c levels and VaMCI for the ε3-ε3 genotype in particular. In other investigations, cognitive performance was decreased in people with diabetes when educational differences were considered;^42^ in our study, we found no association.

In previous studies, attention, executive function, and speed performance showed a decline when assessing for cognitive functions on patients with a continued hypertensive state,^44^ with daily pressure variabilities associated with worse cognition and decreases in global cognitive functions.^45^ Our data did not provide enough evidence to confirm the association between HTN and VaMCI. Similarly, two other studies observed that for every 10 mg increase in small-dense low-density lipoprotein cholesterol (sdLDL-C), there was an accelerated cognitive deterioration.^46^ A high concentration of total cholesterol and LDL-C in late life was associated with a rapid cognitive impairment. On the contrary, another study found that MCI patients had a significant amount of small HDL when compared to the Alzheimer group,^47^ higher midlife HDL-C halve the risk of developing MCI and lowered risk of dementia later in life,^48^ suggesting an association between HDL subclasses and MCI. Whereas the components of MetS were not independently associated with cognitive impairment, MetS as a whole comprised a greater risk of developing accelerated cognitive decline at 10 years,^49^ we did not find such association.

### Limitations and strengths

Limitations included:

- The study population was relatively small and the resulting limited statistical power prevented the direct analysis of the relationship between cardiometabolic risk factors, APOE and MCI.
- Enrolled patients were from a single segment of the city.
- Our sample had a greater proportion of female participants.
- Participants were not matched according to educational level or age.
- Trial Making Test and Digit Span Tests were not included in the protocol due to financial limitation. There was a “floor effect” of the construction and abstract reasoning tests. The neuropsychological battery was developed to be used in Dominicans in New York, and we have implemented it in the Dominican Republic population; the years of education differences in these populations may have cause this floor effect.
- We analyzed only two of the APOE genotype groups with relatively different sizes due to limited proportion in some groups.

Study strengths:

- This is the first study in this population, and no comparisons of APOE 3-3 and 3-4 are available;
- The number of failed cognitive test allowed us to understand the magnitude of the impairment instead of encoding the presence or absence of VaMCI diagnosis.

Hemoglobin A1c and APOE genotypes seem to have an association with the development of VaMCI. Patients with APOE genotypes ε3-ε3 and ε3-ε4 are affected differently by A1c levels. In this sense, tight glucose control must be encouraged in the clinical consults for elderly patients at risk for developing VaMCI, in particular those with an ε3-ε3 and ε3-ε4 genotypes. Routine APOE genotyping and neuropsychological evaluation may be beneficial in the at-risk aged population of Latin-American and Dominican heritage. We found differences that should be explored in future research with larger patient cohorts to uncover further details about the relationship between APOE genotypes, cardiometabolic risk factors and MCI.
